# Supporting employees with mental illness and reducing mental illness-related stigma in the workplace: an expert survey

**DOI:** 10.1007/s00406-022-01443-3

**Published:** 2022-07-22

**Authors:** Bridget Hogg, Ana Moreno-Alcázar, Mónika Ditta Tóth, Ilinca Serbanescu, Birgit Aust, Caleb Leduc, Charlotte Paterson, Fotini Tsantilla, Kahar Abdulla, Arlinda Cerga-Pashoja, Johanna Cresswell-Smith, Naim Fanaj, Andia Meksi, Doireann Ni Dhalaigh, Hanna Reich, Victoria Ross, Sarita Sanches, Katherine Thomson, Chantal Van Audenhove, Victor Pérez, Ella Arensman, Gyorgy Purebl, Benedikt L. Amann, MENTUPP consortium members, Ainslie O’Connor, Andras Szekely, Anthony LaMontagne, Ariel Como, Arilda Dushaj, Asmae Doukani, Azucena Justicia, Birgit A. Greiner, Chris Lockwood, Cliodhna O’Connor, David McDaid, Dooyoung Kim, Eileen Williamson, Eve Griffin, Evelien Coppens, Genc Burazeri, Gentiana Qirjako, Grace Davey, Jaap van Weeghel, Joe Eustace, Joseph Kilroy, Juliane Hug, Kairi Kolves, Karen Mulcahy, Karen Michell, Kristian Wahlbeck, Lars de Winter, Laura Cox, Luigia D’Alessandro, Margaret Maxwell, Nicola Reavley, Peter Trembeczky, Paul Corcoran, Reiner Rugulies, Ruth Benson, Saara Rapeli, Sarah Ihinonvien, Sevim Mustafa, Sharna Mathieu, Stefan Hackel, Tanya King, Ulrich Hegerl, Vanda Scott, Wendy Orchard

**Affiliations:** 1 Centre Fòrum Research Unit, Institute of Neuropsychiatry and Addiction (INAD), Parc de Salut Mar, Barcelona, Spain; 2 Mental Health Research Group, Hospital del Mar Medical Research Institute (IMIM), Barcelona, Spain; 3 PhD Programme, Dept. of Psychiatry and Forensic Medicine, Universitat Autònoma de Barcelona, Bellaterra, Spain; 4 Centro de Investigación Biomédica en Red en Salud Mental (CIBERSAM), Instituto de Salud Carlos III, Madrid, Spain; 5 Institute of Behavioural Sciences, Semmelweis University, Semmelweis Egyetem Magatartástudományi Intézet, Budapest, Hungary; 6 Faculty of Psychology and Psychotherapy, University of Heidelberg, Heidelberg, Germany; 7 National Research Centre for the Working Environment, Copenhagen, Denmark; 8 School of Public Health, University College Cork, Cork, Ireland; 9 National Suicide Research Foundation, Cork, Ireland; 10 Nursing, Midwifery and Allied Health Professionals Research Unit, University of Stirling, Stirling, Scotland; 11 LUCAS, Center for Care Research and Consultancy, Faculty of Medicine, KU Leuven, Louvain, Belgium; 12 European Alliance Against Depression E.V, Leipzig, Germany; 13 Population Health, London School of Hygiene and Tropical Medicine, London, England; 14 Global Public Health, Public Health England, Greenwich, UK; 15 Finnish Institute for Health and Welfare (THL), Helsinki, Finland; 16 Mental Health Center, Prizren, Kosovo; 17 Institute of Public Health, Tirane, Albania; 18 Depression Research Centre of the German Depression Foundation, Department of Psychiatry, Psychosomatic Medicine and Psychotherapy, University Hospital, Goethe University, Frankfurt am Main, Germany; 19 German Depression Foundation, Leipzig, Germany; 20 Australian Institute for Suicide Research and Prevention, Griffith University, Brisbane, Qld Australia; 21 Phrenos Center of Expertise for Severe Mental Illness, Utrecht, the Netherlands; 22 International Association for Suicide Prevention (IASP), Washington, DC USA; 23 Dept. of Psychiatry and Forensic Medicine, Pompeu Fabra University, Barcelona, Spain; 24 Dept. of Psychiatry and Psychotherapy, Ludwig Maximilian University Hospital Munich, Nussbaumstraße 7, Munich, Germany

**Keywords:** Workplace, SME, Depression, Anxiety, Stigma, Mental illness, Expert survey

## Abstract

An expert survey was designed to support the development of a workplace-based multi-country intervention tackling depression, anxiety, and mental illness-related stigma in small- and medium-sized enterprises (SMEs). Academic experts and representatives of SME organisations, specific sector organisations, labour or advocacy groups, and occupational health organisations, were contacted across eight European countries and Australia. The survey comprised closed and open text questions to assess expert opinion about interventions for employees with mental health difficulties, interventions supporting their managers, and anti-stigma interventions. The survey was available in six languages. The online platform Qualtrics was used for data collection. Quantitative data was analysed through descriptive statistics and qualitative data was analysed through thematic analysis. Sixty-five of 146 experts responded, representing a 42% response rate. Results showed only 26.2% of experts agreed that employees could speak openly about mental health issues, and 81.5% of experts indicated a large or medium unmet need for support for employees with mental health issues. Psychoeducational materials, face-to-face workshops and interventions based on cognitive behavioural therapy were ranked most likely to be taken up by employees. Experts rated as most useful for managers’ guidelines on how to act if an employee has mental health issues (67.7%). The greatest number of experts indicated workshops of people with lived experience of mental illness (80.0%) and awareness campaigns (78.5%) were most required to tackle stigma. Responses were consistent between experts from different countries and areas of expertise. Experts in this multinational survey assessed that interventions supporting mental health in the workplace and tackling stigma are greatly needed. A multicomponent intervention with a wide range of materials and tools is supported.

**Supplementary Information:**

The online version contains supplementary material available at 10.1007/s00406-022-01443-3.

## Introduction

It is estimated that 4.4% of the world’s population suffers from depression at any time, and 3.6% from anxiety disorders [1]. Mental illness is projected to have a global economic impact of $6 trillion by 2030 [2]. This includes costs of healthcare, lower productivity due to absenteeism and presenteeism (defined as attending work when ill), [3] and the cost of millions being unable to participate in the workforce. Depression and anxiety represent the leading and sixth cause of disability around the world, respectively [4].

Despite being a major public health concern, there is a reported treatment gap for depression and anxiety, with many receiving no or inadequate treatment [5, 6]. This is related to a range of factors, such as lower country-income level and socio-economic status [7, 8] and male gender [9]. Additionally, mental illness-related stigma creates an important barrier to recovery from depression [10, 11] and anxiety disorders [12, 13]. Stigma can be perceived from the social environment [14], health care professionals [15], and self-labelled stigma can further prevent help-seeking [16]. In the workplace, stigma around mental illness creates a barrier for employment opportunities and promotion [17, 18], while job accommodations to support employees with mental health needs can be met by negative emotional responses from co-workers with stigmatising attitudes [19]. Therefore, reducing stigma is an important strategy for increasing the utilization of mental health services and for supporting people with mental health needs to stay in or return to the workforce. However, reducing stigma can be challenging, and efficacy studies often show mixed results [20, 21], highlighting the need for developing and testing anti-stigma campaigns.

One area of focus for reducing the treatment gap, preventing mental health-related absence, and promoting mentally healthy workplaces are workplace-based mental health interventions [22]. Companies, however, often lack resources to support their employees, particularly small- to medium-sized enterprises (SMEs) [23], which account for 92.8% of the EU workforce [24]. In response to this gap, the Mental Health Promotion and Intervention in Occupational Settings (MENTUPP) project, funded by a European H2020 grant (www.mentuppproject.eu), aims to promote psychological wellbeing and reduce stress and burnout, as well as provide support for clinical depression and anxiety. It also aims to reduce stigma associated with mental health problems. The intervention focuses on SMEs in the construction, the Information and Communications Technology (ICT), and the healthcare sectors in eight European countries and Australia. The intervention countries were chosen in the MENTUPP consortium based on previous experience with multilevel mental health interventions, and/or having experience in mental health promotion and intervention programmes in one of the aforementioned sectors. These sectors were chosen for the increased mental health burden faced by workers in these sectors compared to the average across the workforce. In healthcare, workers already suffered from higher levels of mental illness than the general population, including depression and anxiety [25], before the SARS-CoV-2 pandemic created an additional mental health burden for care workers [26]. There is an increased risk of suicide for employees in both the healthcare and the construction sectors compared to the general workforce [27]. In the construction industry, high suicide rates are related to the context of long working hours, workplace and financial pressure, common substance misuse as a coping strategy, and a negative impact construction work can have on home life [28]. The effect is compounded by high levels of mental health-related stigma in this predominately male environment creating a barrier to help-seeking [29]. However, stigma is also found in the predominately female healthcare sector, even impacting on patient care [30]. The ICT sector, meanwhile, is one of the fastest growing industries in Europe [31] and there is increasing concern over the effects of working in this rapidly changing sector [32, 33]. Working with technology can blur work/life boundaries, increase work pace, and lead to feelings of isolation [34], increasing the risk of mental disorders.

To support the development of tools for the MENTUPP project, an expert survey was developed to supplement gaps identified in the current literature [35]. Where there is a lack of data, expert surveys can inform the best possible approach [36]. The survey, which can be seen in full in Online Resource 1, covered the broad range of aims of the MENTUPP project. Results regarding wellbeing and non-clinical mental health problems and the impact of COVID-19, will be reported elsewhere (Coppens et al. and Cerga-Pashoja et al., in preparation). This article addresses the following research questions related to mental illness and related stigma in the workplace:

1. What support needs of employees with mental health difficulties such as depression, anxiety, self-harm or suicidal thoughts or behaviours are required according to experts, what workplace-based interventions are available, and what are the current gaps?
2. What support needs of managers regarding employees with mental health difficulties such as depression, anxiety, self-harm or suicidal thoughts or behaviours are required according to experts, and what are the current gaps?
3. What are experiences of companies with existing interventions, policies, and best practices for reducing mental illness-related stigma, as assessed by experts, and what are the current gaps?
4. Are there differences in assessments by country region or by area of expertise?

## Methods

### Study sample

Experts from the following categories were invited to participate in the survey: 1) academic experts; 2) representatives of SME organisations; 3) representatives of the construction, healthcare, or ICT sectors; 4) representatives of occupational health association groups, labour groups and advocacy groups. The following exclusion criteria were used: 1) less than 5 years’ experience in their domain; 2) being a member of the MENTUPP consortium; and 3) being < 18 years old. Experts were recruited from the nine countries where the MENTUPP intervention will be trialled: Albania, Australia, Finland, Germany, Hungary, Ireland, Kosovo, the Netherlands, and Spain. Experts were identified through networking, recommendation from other experts, and database and internet searches. The researcher responsible for coordinating the MENTUPP intervention in each country was asked to identify between 5 and 25 experts. A pre-defined quota for each country for experts for each specific category was not included, but each country was requested to invite a wide range of experts to ensure a diverse sample overall, while accounting for the different country sizes.

### Materials

Questions were formulated by researchers from the MENTUPP consortium, with the aim of gathering knowledge to inform the development of the MENTUPP intervention. Specifically for the survey sections in the scope of this paper, the survey was designed to supplement existing knowledge in terms of designing an intervention to improve depression and comorbid anxiety and to reduce mental illness-related stigma in SMEs in the sectors of construction, ICT, and health, focusing the intervention at both employee and supervisor level.

Prior to the development of the survey, a systematic review was carried out, as part of the MENTUPP project, into workplace interventions for depression and anxiety specifically in an SME setting [35]. The review found too few studies in an SME context to draw robust conclusions, but preliminary evidence supported approaches based on cognitive behavioural therapy (CBT). There was a lack of evidence regarding the best format and mode of delivery, but interventions with face-to-face or telephone support appeared to be effective. There was also preliminary evidence in the systematic review supporting a focus on return to work after mental health-related absence. The evidence supporting CBT-based approaches aligns with the more extensive literature regarding interventions for depression and anxiety in larger scale enterprises, although there is greater evidence supporting online formats being effective [37–40]. Overall, our review revealed a lack of data regarding interventions aimed at managers of employees with mental health issues, a lack of studies specific to the construction, health, or ICT sectors, and a lack of evidence for the most effective format and mode of delivery for the intervention. We were also unable to find data regarding what level of mental health support is already available for employees in SMEs in the range of MENTUPP countries. Therefore, questions were formulated to supplement the existing knowledge in terms of:

1. Support for employees with mental health problems such as depression and comorbid anxiety:
  - Understanding what measures of support are available
  - Understanding the current level of unmet need for programmes aimed at preventing and treating mental health difficulties in employees
  - Understanding which of a wide range of tools and materials are already available (ranging from psychoeducational material to face-to-face and online workshops and therapy), and what tools are deemed useful by experts
  - Understanding which materials and tools experts assess are likely to be taken up by employees
2. Currently available support for managers of employees with mental health difficulties:
  - Understanding what skills experts assess managers may already have to manage employees with mental health conditions
  - Which of a wide range of materials and tools is there a need for and which would be useful for managers

Regarding the anti-stigma section, a scoping review as part of the MENTUPP project was undertaken to inform the development of this component. In the scoping review, it was found that specific knowledge regarding interventions in SMEs is largely missing. According to a previous review of 16 interventions targeting mental illness-related stigma in larger enterprises, it was found that anti-stigma interventions could lead to improved employee knowledge and supportive behaviour towards people with mental health difficulties [41]. However, the authors cautioned that further studies with more robust methodology were needed, and these studies were mainly conducted in the public sector with highly educated workers, and thus may not be applicable to SME organisations or other sectors. Another review similarly found a lack of robust studies, and cautioned against generalising from one target group to another [42], while another found that workplace anti-stigma interventions may be enhanced by aiming to change the norms and culture around mental health within an enterprise [43]. Further data came from studies of anti-stigma studies outside of the workplace context. Evidence shows that social contact-based interventions, involving contact with people with a lived experience of mental illness, can be effective short-term [42, 44] but not medium-to long-term [45]. Education interventions may be less effective short-term than social contact-based interventions in adults [44], while group anti-stigma interventions show promise [42], The initial scoping review also revealed evidenced regarding online vs face-to-face interventions for managers, suggesting that online interventions could have the same efficacy as face-to-face interventions if the online intervention is completed [46] and filmed social contact-based interventions may be as effective as face-to-face interventions and be more cost-effective [47]. Therefore, the scoping review showed preliminary evidence that anti-stigma interventions can be effective in a workplace setting but there were knowledge gaps regarding what intervention components and delivery formats were suitable for a workplace setting, and no evidence specifically in an SME setting. Based on this, the questions were devised to understand the following:

1. Understanding levels of stigma
  - Understanding what levels of stigma employees with mental health issues currently face
  - Understanding the extent that workplaces have policies in place to reduce discrimination regarding mental health issues
  - Understanding the common attitudes of employees and managers in revealing/being open to mental health issues
  - Understanding the experts’ perception of the risks and benefits of employees being open about their mental health problems
2. Activities to reduce stigma
  - Understanding the level of need for a range of anti-stigma interventions (e.g. online materials, counselling, workshops with people with lived experience)
  - Understanding experts’ assessment of likely manager attitudes towards anti-stigma programmes
  - Understanding barriers to implementing anti-stigma activities
  - Understanding which anti-stigma activities experts are aware of in each country

Once the questions were formulated, based on the above, the survey was then piloted within the MENTUPP consortium and the final version approved by all members. Closed and open questions were used to obtain both quantitative and qualitative data. Given the heterogeneity of experts and the broad lack of a specific mental health background, clinical mental health terms such as major depressive disorder were not used. Instead, experts were asked about “mental health difficulties or issues such as depression, anxiety, self-harm and suicidal thoughts or behaviour”. The scope of the MENTUPP project in terms of clinical mental health issues is limited to depressive and comorbid anxiety disorders and suicide prevention. Therefore, our questions were focused also on these areas and other mental health difficulties, such as psychosis or substance use disorders, were not included. Due to the diversity of experts, the response category “I don’t know” was added to a standard Likert scale to avoid participants answering items outside their realm of expertise and thus introducing bias.

The survey and informed consent document were prepared in English and local language versions were additionally provided for Albania, Germany, Hungary, Kosovo, the Netherlands, and Spain. The English versions of the informed consent document and survey were uploaded into the Qualtrics platform for online surveys (www.qualtrics.com). Local language versions were administered via Word or paper format, and the results translated into English and entered into Qualtrics by each country’s lead researcher. The Qualtrics platform was set so that all quantitative data answers were a required field, meaning there was no missing data in completed responses.

### Survey overview

The survey focused in total on seven topics, of which the following three form the basis of this paper:

1. General information about the participants and their backgrounds
2. Perceived effectiveness of interventions:
  - Support for employees with mental health difficulties.
  - Support for the managers of employees with mental health difficulties.
3. Anti-stigma activities, which comprised two sub-sections:
  - Perceived current levels of stigma.
  - Anti-stigma activities.

The topics covered by the survey not presented here were: 1) Workplace activity with regards to promoting psychological wellbeing and (non-clinical) mental health; 2) Impact of COVID-19 on employees’ mental health; 3) Gender-specific needs; and 4) Acceptability of workplace-based interventions.

### Procedure

The survey was sent out by the lead researcher in each country to experts between 15/09/2020 and 5/10/2020. Participation was voluntary and only proceeded once informed consent was obtained. The survey was anonymous and estimated to take approximately 20 to 50 min to complete. Participants could save their answers and resume their survey across multiple sessions if desired. To maximise engagement, multiple general reminders were sent to encourage participants to complete the survey.

### Analyses

Descriptive statistics were used to analyse the closed question survey responses. Percentage responses were calculated for each item and are shown in the results. Additionally, the median response and the interquartile range (IQR; the distance between the 25^th^ and the 75^th^ percentiles) were calculated to determine the levels of agreement on the items, using the ordinal data from the Likert scales with the category “I don’t know” omitted.

Due to the heterogeneity of experts, sub-analyses were conducted to determine whether a country region or area of expertise significantly affected results. The sub-analysis by classification of countries was based on geographical area and resulted in two country groups: (1) Western Europe & Australia: Australia, Finland, Germany, Ireland, Netherlands, Spain; and 2) Central & Eastern Europe: Hungary, Albania, Kosovo [48]. For the sub-analysis regarding area of expertise, the experts were grouped as follows: 1) experts representing the construction, health or ICT sectors; (2) academic experts; (3) experts representing SME organisations; and (4) representatives of labour or advocacy groups or occupational health specialist association groups. Non-parametric *χ*^2^-tests (Chi-squared tests) were carried out to assess between-group differences. The significance level was set at *p* < 0.05.

Thematic analysis was used to analyse the open text answers [49] by two independent researchers using Dedoose® software following an inductive approach, i.e. the data itself formed the structure of the analysis rather than using a pre-existing framework. Discrepancies were resolved in a consensus meeting.

## Results

Across the 9 countries, 146 experts were invited to participate, of which 65 completed the survey, representing a response rate of 42%. There was a slight majority of male participants (56.9%). The age group 40–49 was most prominent (35.4% of total), but experts from all age groups (ranging from 20–30 to 70 +) participated. The distribution by country was uneven, with 24.6% of experts based in Albania, while Germany, Ireland and Australia had fewer than five experts each and represented 6.2%, 4.6% and 3.1% of the total, respectively. All expert types were represented, with representatives of the construction, health or ICT sectors, and then academic experts, best represented (49.2% and 23.1%, respectively). The breakdown of expert type by country can be seen in Online Resource Table 1, and in Online Resource Fig. 1. In terms of specific areas of expertise, 47.7% of experts included the healthcare sector as one of their areas of expertise, compared to 24.6% for the ICT sector and 13.8% for construction, while 18.5% had expertise in mental health in SMEs and 16.9% in SMEs in general. A complete overview can be seen in Table 1.

**Table 1:** Participant characteristics

Participant characteristic	Frequency (%)
**Gender**	
Female	26 (40)
Male	37 (56.9)
Other	2 (3.1)
**Age**	
20–29 years	3 (4.6)
30–39 years	15 (23.1)
40–49 years	23 (35.4)
50–59 years	14 (21.5)
60–69 years	7 (10.8)
70 + years	3 (4.6)
**Country**	
Albania	16 (24.6)
Australia	2 (3.1)
Finland	6 (9.2)
Germany	4 (6.2)
Hungary	10 (15.4)
Ireland	3 (4.6)
Kosovo	8 (12.3)
Spain	9 (13.8)
The Netherlands	7 (10.8)
**Type of Representative**	
Representative of construction, health or ICT sector	32 (49.2)
Academic expert	15 (23.1)
Representative of SME group	4 (6.2)
Representative of labour, occupational health or advocacy group	5 (7.7)
Other	9 (13.8)
**Years of expertise**	
5–10 years	21 (32.3)
11–20 years	27 (41.5)
20 + years	17 (26.2)
**Area of expertise (more than one option may be selected)**	
SMEs	11 (16.9)
Mental health in SMEs	12 (18.5)
Construction sector	9 (13.8)
Healthcare sector	31 (47.7)
ICT sector	16 (24.6)
General and not related to any of these sectors (e.g. academic expert or representative of non sector-specific organisation)	11 (16.9)

### Support needs of employees with mental health difficulties

The majority of experts believed that a variety of support measures for employees with mental health difficulties were not widely available, with 67.7% answering that support provided directly in the workplace was available either “to a small extent” or “not at all”. For the other three questions about support measures, more than half of the experts answered “to a small extent” or “not at all” (52.3% for support provided by a third party, 56.9% for support provided by health insurance through the business, and 67.6% for support from labour organisations). The full results can be seen in Table 2.

**Table 2:** Currently available measures of support for employees with mental health difficulties by *n* and % of experts

Measures of support	To a large extent (4)	Somewhat (3)	To a small extent (2)	Not at all (1)	Don’t know	M (IQR)
Support supplied directly within the workplace	6 9.2%	12 18.5%	26 40%	18 27.7%	3 4.6%	2 (2)
Support supplied by a third party	10 15.4%	19 29.2%	23 35.4%	11 16.9%	2 3.1%	2 (1)
Support provided by health insurance through the business	4 6.2%	17 26.2%	21 32.3%	16 24.6%	7 10.8%	2 (2)
Support from labour organisations	4 6.2%	11 16.9%	22 33.8%	22 33.8%	6 9.2%	2 (2)

Key. M: Median; NA: Not applicable; IQR: Inter-Quartile Range

Next, experts were asked for their opinion on a wide range of materials and tools (see Table 3). No materials or tools were assessed by a majority of experts as being currently available either “to a large extent” or “somewhat”, while all were judged useful by a majority of experts, except for “interventions based on other therapies (as specified by the respondent)”, which were deemed useful by 30.8%. Those judged by the largest majority as useful were “frameworks to guide planning return after mental-health related absence” (73.8%), “information about depression or anxiety and how to cope” (72.3%), “frameworks to guide addressing mental health issues with employee” and “frameworks to guide accessing health services” (both 70.8%). Finally, experts were asked to rank the five tools and materials they thought were most likely to be taken up by staff experiencing mental health difficulties. The tools or materials deemed most likely to be taken up by staff were: (1) information about depression or anxiety and how to cope; (2) online workshops on detecting and managing depression and/or anxiety; and (3) interventions based on CBT. Experts were also asked to rate the level of unmet need for programmes to prevent and treat mental health difficulties at the workplace. This was rated as high by 29 experts (44.6%), medium by 24 experts (36.9%) and low by 6 experts (9.2%). No experts rated this as “no need”, while 6 (9.2%) responded “don’t know” (see Online Resource Fig. 2).

**Table 3:** Tools and materials available for employees with mental health difficulties by *n* and % of experts

Type of tool/material	To what extent are these tools and materials available for employees with mental health difficulties? (% expert responses)						Would the following materials be useful for employees?		Most likely to be taken up by staff
	To a large extent (4)	Somewhat (3)	To a small extent (2)	Not at all (1)	Don’t know	Median (IQR*)	Yes Frequency (%)	No Frequency (%)	Ranking
Information about depression or anxiety and how to cope	9 13.8%	10 15.4%	17 26.2%	23 35.4%	3 4.6%	2 (2)	47 72.3%	6 9.2%	1
Face-to-face workshops on detecting and managing depression and/or anxiety	4 6.2%	10 15.4%	18 27.7%	25 38.5%	5 7.7%	2 (2)	41 63.1%	9 13.8%	2
Interventions based on cognitive behavioural therapy	2 3.1%	10 15.4%	17 26.2%	26 40%	7 10.8%	2 (1)	45 69.2%	5 7.7%	3
Peer support interventions	7 10.8%	9 13.8%	23 35.4%	17 26.2%	6 9.2%	2 (2)	45 69.2%	4 6.2%	4
Online workshops on detecting and managing depression and/or anxiety	2 3.1%	11 16.9%	16 24.6%	24 36.9%	9 13.8%	2 (2)	36 55.4%	16 24.6%	5*
Online tools to detect and manage depression and/or anxiety	4 6.2%	13 20%	18 27.7%	22 33.8%	5 7.7%	2 (2)	40 61.5%	12 18.5%	5*
Frameworks to guide addressing mental health issues with employee	7 10.8%	9 13.8%	14 21.5%	25 38.5%	6 9.2%	2 (2)	46 70.8%	4 6.2%	7
Interventions based on mindfulness or relaxation techniques	5 7.7%	16 24.6%	17 26.2%	19 29.2%	3 4.6%	2 (2)	44 67.7%	6 9.2%	8
Frameworks to guide planning return after mental health-related absence	8 12.3%	10 15.4%	15 23.1%	21 32.3%	8 12.3%	2 (2)	48 73.8%	2 3.1%	9
Information about suicide and how to access help	3 4.6%	11 16.9%	16 24.6%	30 46.2%	3 4.6%	1.5 (1)	38 58.5%	10 15.4%	10
Frameworks to guide accessing health services	7 10.8%	10 15.4%	27 41.5%	13 20%	5 7.7%	2 (1)	46 70.8%	4 6.2%	11
Interventions based on other therapies (as specified by the respondent)	1 1.5%	7 10.8%	6 9.2%	8 12.3%	15 23.1%	2 (2)	20 30.8%	6 9.2%	NA

Key. M: Median; NA: Not applicable; IQR: Inter-Quartile Range

*These responses were ranked equally in 5^th^ place

### Support needs of managers of employees with mental health difficulties

There was consensus among experts that managers currently lack the knowledge and skills to detect mental illness in an employee or to have a conversation about this (in both cases, 69.2% of experts rated this “to a small extent” or “not at all”), or to make adjustments to facilitate job retention or return to work for employees affected by mental illness (63.0% rated this “to a small extent” or “not at all”; see Online Resource Table 2).

All the proposed materials aimed at supporting supervisors of employees with mental health difficulties were assessed as needed “to a large extent” or “somewhat” by a majority of experts (see Online Resource Table 3). The materials most highly rated were guidelines on what to do if an employee is experiencing mental health issues (assessed as needed “to a large extent” or “somewhat” by 80.0% of experts), guidelines on handling an employee’s return following mental health-related absence (78.4%), and information about depression or anxiety and how to cope (73.9%). Experts were then asked to rate the usefulness of these resources. The material rated most useful for supervisors were guidelines on what to do if an employee is experiencing mental health issues (rated useful by 67.7% of experts), followed by guidelines on handling an employee’s return to work following mental health-related absence, and face-to-face workshops with healthcare professionals (both rated useful by 64.6% of experts).

### Interventions, policies, and best practices for reducing mental illness-related stigma

While 26.2% of experts agreed or strongly agreed with the sentence “employees can speak openly about their work stress, burnout, feelings or mental health problems”, 46.1% either disagreed or strongly disagreed, 18.5% were neutral and 8.2% rated “don’t know” (see Online Resource Table 4).

Regarding the extent to which workplaces have policies on sharing information about employee’s mental health difficulties, and policies to protect employees against discrimination and bullying due to mental illness, in all cases a majority of experts (58.5% and 63.1%, respectively) felt these are generally available only “to a small extent” or “not at all”. Only 32.3% of the experts assessed that workplaces “to a large extent” or “somewhat” have a visible approach to reduce bullying and discrimination related to mental health difficulties in the workplace (see Online Resource Table 5).

**Fig. 1:**
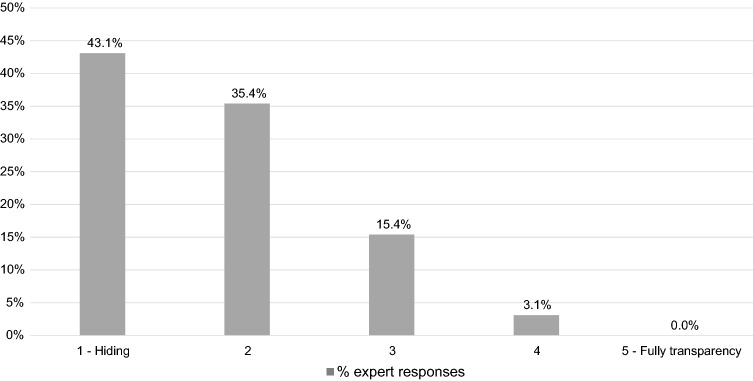
Expert assessment of most common attitude of employees towards openly expressing mental health problems in the workplace

**Fig. 2:**
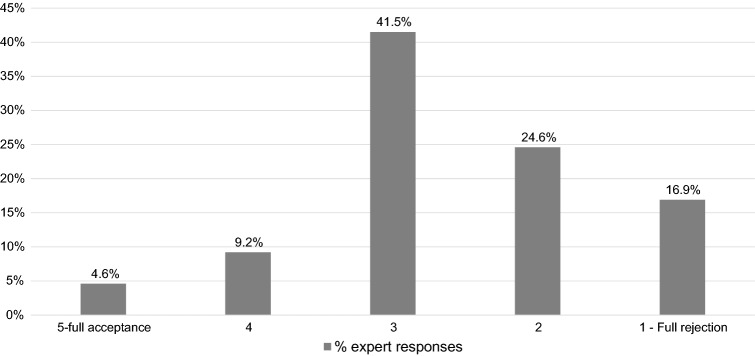
Expert assessment of most common attitude of managers towards employees openly expressing mental health problems in the workplace

Most experts thought the most common employee attitude towards mental health issues was to hide them completely or to some degree (78.5%), while no expert felt that the most common attitude was to be fully open (see Fig. 1) In terms of what experts perceived was the most common attitude of managers towards employees openly expressing mental health problems, only a minority felt managers would be fully or partially accepting (4.6% and 9.2%, respectively), whereas 16.9% felt they would fully reject an employee (see Fig. 2).

Experts were asked an open text question about the most common risks and benefits regarding employees openly expressing their mental health problems. The two most common risks identified by experts were concern about job loss through dismissal (26.2% of experts) and stigmatization (24.6%; see Online Resource Fig. 3). The two most common benefits were getting support from colleagues or managers (24.6% of experts), and colleagues and managers being more understanding (18.5%; see Online Resource Fig. 4).

**Fig. 3:**
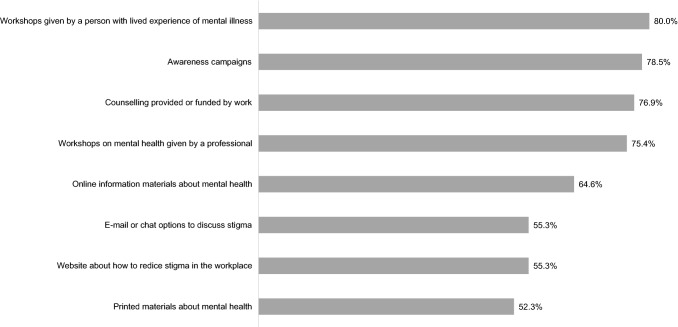
Anti-stigma materials: % rated as needed "To a large extent" or "somewhat"

All proposed anti-stigma activities to reduce mental health-related stigma were assessed as needed to “to a large extent” or “somewhat” by most experts (see Fig. 3). The most highly rated activities were workshops on mental health given by a person with lived experience of mental illness (rated “to a large extent” or “somewhat” by 80.0% of experts), awareness campaigns (79%), and mental health counselling (77%).

Finally, experts were asked about the potential benefits of anti-stigma programmes. Most experts felt that managers would agree “to a large extent” or “somewhat” that anti-stigma programmes have a positive impact (75.3%), anti-stigma programmes can increase wellbeing (73.8%), and anti-stigma programmes can increase productivity (70.8%; see Online Resource Table 6 for results).

In response to an open text question regarding barriers towards implementing anti-stigma activities, the most commonly perceived barriers were stigma-related shame and also the lack of knowledge about their importance (both mentioned by 16.9% of experts; see Online Resource Fig. 5 for full results).

Additionally, 21 experts (32.3%) provided information about anti-stigma activities conducted in the country where they were based (see Online Resource Table 7).

### Differences in assessments due to country region or area of expertise

The sub-analysis by country region included 31 experts from Western Europe & Australia, compared to 34 from Central and Eastern Europe. There were significant differences in responses in only five items. Policies on sharing information about employees’ mental health to ensure their privacy is protected were slightly more prevalent in Western Europe & Australia, with significantly more experts answering that these were available “to a small extent”, compared to “not at all” in Central and Eastern European countries (*p* = 0.047). Face-to-face workshops for employees (p = 0.048) were rated as more useful by Central and Eastern European countries. Interventions based on CBT, as well as interventions based on mindfulness and other relaxation techniques, were ranked significantly higher in terms of how likely they were to be taken up by staff by Western Europe and Australia (*p* = 0.036 and *p* = 0.033, respectively). Face-to-face workshops for managers were judged to be needed to a significantly larger extent in Central & Eastern Europe (*p* = 0.010), and guidelines for managing presenteeism were judged more useful in this country group (*p* = 0.032) (see Online Resource 2).

Regarding anti-stigma programmes, only 14.7% of respondents from Central and Eastern European countries named a programme implemented locally compared to 51.6% of respondents from Western Europe and Australia. At least one anti-stigma programme was referenced in all countries except for Hungary, and experts from Finland referenced the largest range of programmes (see Online Resource Table 6).

The sub-analysis for response by type of expert showed a large degree of consensus despite the heterogeneity between experts, with no significant difference between type of experts in all but four items (see Online Resource 3).

## Discussion

The present study shows overall consistent responses from a range of experts regarding a perceived lack of workplace-based resources and interventions for supporting employees with mental illness and their managers, and for tackling mental illness-related stigma. Responses were also consistent regarding how to meet this need. For employees, the highest rated materials were psychoeducational materials, face-to-face workshops and interventions based on CBT, which are in line with data from recent reviews in both SMEs and larger enterprises [35, 37, 38, 40, 50]. To support managers, the interventions assessed to be the most useful included information about how to cope with depression or anxiety in employees, as well as guidelines on what to do if an employee is experiencing mental health issues, and guidelines on supporting an employee’s return to work following a mental health-related absence. Our results are aligned with the results of a review in enterprises of all sizes, which found that training managers in workplace mental health can have a positive effect on their knowledge, attitudes and self-reported behaviour when supporting an employee with a mental health issue, although there was limited data on whether this translated to reduced psychological distress in employees [46]. The inclusion of these aspects in the MENTUPP intervention should be able to provide data in the future regarding the impact of these measures in improving employee mental health outcomes.

Regarding our experts’ assessment of the need for guidelines on supporting an employee’s return to work following a mental health-related absence, this concurs with previous evidence that SMEs have a lower capacity than larger companies to manage this successfully [51]. Indeed, in our study, only 37% of experts were confident that managers “to a large extent” or “somewhat” currently have the skills to manage an employee’s return to work following mental health-related absence. The results are striking when contrasted with the legal requirements in many countries that require reasonable adjustments in the workplace to be made for employees with mental illness [52]. Psychosocial working conditions have been shown to affect return to work [53], and an accommodating workplace is an important part of ensuring that return to work programmes are effective [54]. A previous expert consensus study has provided guidelines to assist managers in this challenging area [55], including creating a clear return to work plan with on-the-job support and mentoring schemes amongst many others. However, the present study and previous research indicate that best practice is seldom implemented in return to work practices following mental illness [56], and could, therefore, be an important aim for any workplace intervention looking at supporting employees with mental health difficulties.

There was a large degree of consensus among experts regarding the need for mental health-related anti-stigma programmes and which strategies could best fulfil them. Experts perceived that employees usually try to conceal mental illness, with the most common underlying reasons as assessed by the experts being the fear of job loss, stigmatisation, rejection by colleagues and discrimination in general. These results are unsurprising given the high levels of stigma surrounding mental health in general [10, 12, 15]. Meanwhile, the highest rated strategy to combat stigma was workshops run by people with lived experience of mental illness, a finding which is consistent with previous research in non-occupational environments [57], although further evidence is needed medium- to long-term [45]. Most of the experts reported that there are few or no mental health-related activities addressing stigma in the workplace, which is in accordance with the scientific literature in companies of all sizes [43]. Of note, a majority of experts (67.7%) were unable to reference an anti-stigma programme in the country where they are based, a figure which was proportionally higher in experts from Central and Eastern Europe. Among the experts that were aware of anti-stigma activities, benefits were noted, such as increased support and understanding, facilitating problem-solving and help-seeking and creating flexible workplace conditions adjusted to the employees’ needs. However, this was balanced with reported concerns regarding implementation, such as a lack of resources, hesitation on the part of employees, or concerns that the workplace is not an appropriate setting. Stigma may have a disproportional impact in hindering the uptake of mental health interventions in SMEs, where there may be no dedicated human resource or occupation health function [58, 59]. Given the increased difficulties of SMEs in implementing health promotion programmes in general [60], these concerns should be given special attention when designing anti-stigma interventions in an SME context.

In terms of implementation, experts rated face-to-face workshops as more useful than online workshops for both employees with mental health issues and their managers. Face-to-face workshops were also rated as more likely to be taken up by staff compared to online workshops. Similarly, experts rated face-to-face anti-stigma interventions highly (e.g. counselling, workshops given by expert-through-experience, workshops given by a professional). Despite our survey being carried out several months into the COVID-19 pandemic, which has forced many interventions to be implemented online, these results may reflect experts’ greater familiarity with traditional face-to-face approaches. The results are in contrast with research showing that online modalities can be effective and reduce costs [50, 61], which may be especially important in the SME context where few resources are usually available, but may reflect that more direct, human contact is still preferred when both options (hypothetically) are available.

A discrepancy was noted between the responses from experts about what interventions were most likely to be taken up by staff, and the ratings on usefulness. For example, frameworks designed to guide accessing health services were rated the least likely to be taken up by staff despite being rated useful by 70.8% of experts. This highlights the need for interventions to be planned not just based on what material is useful, but also ensure they are acceptable for employees in a workplace context. This is especially important in SMEs where take up of health promotion programmes is generally lower than in larger enterprises [60].

The consistency in responses across country groups was surprising given that all the countries in the Western Europe & Australia group are classed as developed economies [62], while in the Central & Eastern Europe group, two of the three countries are classed as economies in transition, with presumably fewer resources to dedicate to mental health and combatting stigma [63, 64]. This consistency in responses was also seen across experts coming from a wide range of fields of expertise and different geographical areas. Given that we were looking for specific expertise in certain sectors and/or the SME workplace, we assumed a small pool of potential experts in each country and designed the sampling strategy to gain access to as broad a range of experts as possible, to ensure that the experts adequately reflected the field of potential and relevant responses. The expert response rate of 42% was low, although appears similar to other web-based expert surveys [65, 66]. Some countries did not meet the minimum target for five experts. However, the impact of this seems to be mitigated by the consistent results from a heterogenous group (in terms of expertise and geographical location), and our results clearly demonstrate a shared unmet need, which supports the feasibility of designing an intervention which can be used across multiple contexts.

Results from experts representing the three sectors of construction, health, and ICT sectors, were consistent with responses from the experts with more general expertise. The results of the MENTUPP pilot trial [67] will show whether the intervention is similarly effective across sectors, but the consistency of expert responses suggests that our findings may be applicable in other workplace settings, although further research is needed.

The strengths of this expert survey include that it was conducted in nine countries in different geographical, political, cultural, and economic regions, and its translation into six languages. Furthermore, a diverse range of experts were consulted, and despite their individual roles, sectors or cultures, answers were largely consistent between expert groups. Moreover, the mix of closed and open questions allowed a quantitative assessment supplemented by detailed qualitative data. However, there were some important limitations. Expert consultations are low in the hierarchy of evidence [36, 68]. Nevertheless, for the specific objectives of this survey, it has provided useful guidance in specific areas where scientific literature is lacking. As previously mentioned, the response rate at 42.0% was low and the distribution of participants per country was uneven, although our sub-analysis of country group and expert area showed few differences. Finally, the translation of the survey and answers may have introduced bias.

In conclusion, the survey results demonstrate that experts, despite their diversity in terms of country and experience, largely come to a very similar assessment regarding a lack of current tools, materials and support for employees and managers to be able to cope with mental health difficulties in the workplace. Similarly, most experts agreed that employees often hide their mental health difficulties due to stigma, and that appropriate workplace-based anti-stigma programmes are needed. The results of this expert survey provide valuable information which adds to the limited empirical evidence available and inform the approach taken within the MENTUPP programme regarding clinical mental health problems and stigma.

## Supplementary Information

Below is the link to the electronic supplementary material.

Supplementary file1 (DOCX 27 KB)

Supplementary file2 (DOCX 32 KB)

Supplementary file3 (DOCX 33 KB)

Supplementary file4 (DOCX 41 KB)

Supplementary file5 (DOCX 46 KB)
