# Supplementary material for: Supporting employees with mental illness and reducing mental illness-related stigma in the workplace: an expert survey
Source: Eur Arch Psychiatry Clin Neurosci. 2022 Jul 22;273(3):739–53. doi: 10.1007/s00406-022-01443-3 (PMC9305029; doi:10.1007/s00406-022-01443-3)
Supplement: Supplementary file 1 — Supplementary file1 (DOCX 27 KB) [file 406_2022_1443_MOESM1_ESM.docx]

**Online Resource 1. Expert Survey in Full.**

**Supporting employees with mental illness and reducing mental illness-related stigma in the workplace: an expert survey**

Bridget Hogg^1,2,3,4^, Ana Moreno-Alcázar^1,2,4^, Mónika Ditta Tóth^5^, Ilinca Serbanescu^6^, Birgit Aust^7^, Caleb Leduc^8,9^, Charlotte Paterson^10^, Fotini Tsantilla^11^, Kahar Abdulla^12^, Arlinda Cerga-Pashoja^13,14^, Johanna Cresswell-Smith^15^, Naim Fanaj^16^, Andia Meksi^17^, Doireann Ni Dhalaigh^9^, Hanna Reich,^18,19^ Victoria Ross^20^, Sarita Sanches^21^, Katherine Thomson^22^, Chantal Van Audenhove^11^, Victor Pérez^,1,2,4,23^, Ella Arensman^8,9,20,22^, Gyorgy Purebl^5^*, Benedikt L. Amann^1,2,4,23,24^ and the MENTUPP consortium

1.Centre Fòrum Research Unit, Institute of Neuropsychiatry and Addiction, Parc de Salut Mar, Barcelona, Spain

2.Mental Health Research Group, Hospital del Mar Medical Research Institute (IMIM), Barcelona, Spain

3.PhD Programme, Dept. of Psychiatry and Forensic Medicine, Universitat Autònoma de Barcelona, Bellaterra, Spain

4.Centro de Investigación Biomédica en Red en Salud Mental (CIBERSAM), Madrid, Spain

5.Institute of Behavioural Sciences, Semmelweis University, Budapest, Hungary

6.Faculty of Psychology and Psychotherapy, University of Heidelberg, Heidelberg, Germany.

7.National Research Centre for the Working Environment, Copenhagen, Denmark

8.School of Public Health, University College Cork, Cork, Ireland

9.National Suicide Research Foundation, Cork, Ireland

10.Nursing, Midwifery and Allied Health Professionals Research Unit, University of Stirling, Stirling, Scotland

11. LUCAS, Center for Care Research and Consultancy, Faculty of Medicine, KU Leuven, Belgium

12.European Alliance Against Depression e.V., Leipzig, Germany

13.Population Health, London School of Hygiene and Tropical Medicine, London, England

14.Global Public Health, Public Health England, United Kingdom

15.Finnish Institute for Health and Welfare (THL)

16.Mental Health Center, Prizren, Kosovo

17.Institute of Public Health, Tirane, Albania.

18.Depression Research Centre of the German Depression Foundation, Department of Psychiatry, Psychosomatic Medicine and Psychotherapy, University Hospital, Goethe University, Frankfurt am Main, Germany

19.German Depression Foundation, Leipzig, Germany

20.Australian Institute for Suicide Research and Prevention, Griffith University, Queensland, Australia

21.Phrenos Center of Expertise for severe mental illness, Utrecht, the Netherlands

22.International Association for Suicide Prevention (IASP), Washington DC, USA

23.Dept. of Psychiatry and Forensic Medicine, Pompeu Fabra University Barcelona, Spain

24.Dept. of Psychiatry and Psychotherapy, Ludwig Maximilian University Hospital Munich, Nussbaumstraße 7, Munich, Germany

*Corresponding author. E-mail: purebl.gyorgy@gmail.com

**Online Resource 1. Expert survey in full.**

**A) General questions about you and your background**

**1. Please indicate your gender:**

1. Male
2. Female
3. Other/prefer not to say

**2. Please indicate your age:**

1. 20-29 years old
2. 30-39 years old
3. 40-49 years old
4. 50-59 years old
5. 60-69 years old
6. 70 years or older

**3. Please indicate the country that you will refer to when providing your answers: (Drop-down list:** *Albania. Australia. Finland. Germany. Hungary. Ireland. Kosovo. The Netherlands. and Spain)*

**4. Please mark the statement that best describe your expertise.**

1. I represent an organisation representing the construction. health or information and communication technologies (ICT) sector

Please briefly describe what type of organisation that is____________________

1. I am an academic expert
   Please briefly describe your academic background and your area of research with relation to mental health at work:______________________________________
2. I represent an organisation providing services for SMEs or representing a group of SMEs
   Please briefly describe what type of organisation that is_______________________
3. I am a labour group. occupational health specialist association group. or advocacy group representative
   Please describe shortly the type of organisation you represent____________
4. Other. please state: ­­­­­­­­­­­­­­­­­­­­__________________

**5. How many years of previously mentioned expertise do you have?**

5-10 years

11-20 years

+20 years

**6. Please indicate if you have expertise in any specific area listed below (multiple boxes can be checked):**

1. SMEs
2. Mental health in SMEs
3. Construction industry
4. Health care sector
5. IT and communication
6. My experience is general and not related to any of these sectors

*Please provide us with your valuable opinion and answer this survey based on your relevant occupational experiences as you have detailed above. Please always respond to the questions in terms of the country you are based in (as indicated above). and any specific sector you represent. if applicable.*

**B. Workplace activity**

**Please respond to the following questions in the context of the period before the current pandemic. There will be space at the end of this section to add comments related to the COVID-19 pandemic.**

1. **Workplace activities**
   1. **In your opinion. to what extent does the average workplace** (*To a large extent/Somewhat/ To a small extent/Not at all/ Don’t know)*
      1. Create mentally healthy workplaces by. for example. providing flexible and supportive working conditions and/or avoiding stressful working conditions. such as long working hours. excessive workload or poor supervisory support
      2. Have a strategic and coordinated organisational approach to promote employees' mental wellbeing
      3. Carry out needs assessments among employees to inform an organisational approach to promote mental wellbeing.
      4. Provide training for managerial/HR staff on promoting wellbeing in the workplace
      5. Provide psychological support services to employees (for example. counselling support and stress management training)
      6. Have a strategic and coordinated organisational approach to reduce stigma related to mental health problems.

**Comments:** Please add any comments you would like to make with regard to workplace activities that address the promotion of mental wellbeing and the prevention. detection and management of stress. burnout. depression or anxiety:_______________(*open text*)

1. **Access to information/tools/advice**
   1. To what extent do you think workplaces would benefit from/ would like/ more/require increased availability of information. about the following topics (*To a large extent/Somewhat/To a small extent/Not at all/Don’t know)*
      1. How to create mentally healthy working conditions
      2. Factors contributing to work stress and burnout
      3. How to establish policies about creating mentally healthy workplaces
      4. How to carry out a needs assessment to inform an organisational approach to promoting wellbeing
      5. How to strengthen people management skills among senior staff/HR staff in order to detect and handle mental health problems
   2. Do workplaces have any other needs when it comes to improving the promotion of employee mental wellbeing and the prevention of employee stress. burnout. depression and/or anxiety? *(open text_____________)*
2. **Experience with existing tools/interventions and challenges**
   1. Based on your experiences what methods/policies/interventions. if any. work well and are accepted in terms of promoting employee mental wellbeing and preventing. detecting. and managing employee stress. burnout. depression. or anxiety? *(open text)*_____________________________________________
   2. Can you suggest up to 5 key barriers that you are aware of or have experienced when implementing methods/policies/interventions aimed at promoting employee mental health?

1.____________________________

2.____________________________

3.____________________________

4.____________________________

5.____________________________

- 1. Can you suggest up to 5 key things that you are aware of which have helped when implementing methods/policies/interventions aimed at promoting employee mental health?

1.____________________________

2.____________________________

3.____________________________

4.____________________________

5.____________________________

1. **Consequences**
   1. Based on your experiences to what extent do you perceive the following business outcomes to be related to poor employee mental health? *(To a large extent/Somewhat/To a small extent/Not at all/Don’t know)*
      1. Absenteeism
      2. Presentism in terms of lower productivity
      3. Difficulties in returning to work following absence
      4. Job turnover (employees with poor mental health resigning or being dismissed)
      5. Other _________
2. **COVID-19: Impact of the COVID-19 pandemic on mental health at work**
   1. **To what extent do you think COVID-19 has impacted on: (***have increased/stayed the same/have decreased/don’t know)*
      1. levels of job stress and burnout
      2. levels of depression. anxiety and/or suicidal thoughts or behaviour
      3. the capacity of workplaces to promote employee wellbeing
      4. the capacity of workplaces to support employees with mental health conditions
      5. the capacity of business owners/managers to look after their own mental health needs
      6. Stigma (negative attitudes/behaviours around mental health issues) surrounding mental health issues
      7. Do you know of any challenges specific to SMEs? ___________________
   2. Please provide further details on the impact of COVID-19 on your area of expertise.*(open text:___________________________________)*

**C. Supporting the individual employee with mental health needs**

**Please respond to the following questions in the context of the period before the current pandemic.**

1. **Support for employees**
   1. To what extent are the following measures of support available for employees showing signs of mental health difficulties such as depression. anxiety or self-harm/suicidal thoughts or behaviour?: *(To a large extent/Somewhat/To a small extent/Not at all/Don’t know)*
      1. support for mental health issues supplied directly within the workplace
      2. occupational health support supplied by a third party
      3. support provided by healthcare insurance organised through the business
      4. support from labour organisations
      5. Other. please state:________________
   2. How do you estimate the current level of unmet need for programs aimed at preventing and treating mental health difficulties in employees? *(High/Medium/Low/No need/ Don’t know)*
   3. To what extent are the following materials/tools available. within the workplace. for employees with mental health issues such as depression. anxiety or suicidal thoughts or behaviour? (*To a large extent/Somewhat/To a small extent/Not at all/Don’t know. (and tick box on side - would this be useful?)*
      1. materials providing information about depression or anxiety and how to cope
      2. materials providing information about suicide and how to access help
      3. face-to-face workshops on detecting and managing depression and/or anxiety
      4. online workshops on detecting and managing depression and/or anxiety
      5. online tools to help to detect and manage depression and/or anxiety
      6. interventions based on cognitive behavioural therapy. to help change negative thoughts and behaviours
      7. interventions based on mindfulness or relaxation techniques
      8. interventions based on other therapies (please specify_____)
      9. peer-support interventions
      10. framework to guide addressing the issue with their employee
      11. framework to guide accessing health services
      12. framework to guide planning a return to work following mental-health related absence (recovery)
      13. Other. please state_____________
   4. Based on your knowledge or experience. can you suggest what types of tools are most likely to be taken up by staff in your sector who are experiencing mental health problems? Please list in order of preference:

1.

2.

3.

4.

5.

1. **Support for managers**
   1. In your experience. to what extent do supervisors have the knowledge and skills to: *(To a large extent/Somewhat/To a small extent/Not at all/Don’t know)*:
      1. detect a mental health condition in an employee
      2. have a conversation with an employee about their mental health condition
      3. make adjustments to facilitate job retention or return to work
   2. To what extent do you think supervisors need the following materials/tools? *(To a large extent/Somewhat/To a small extent/Not at all/Don’t know and tick box on side - would this be useful?)*
      1. Materials providing information about depression and anxiety
      2. Materials providing information about suicide and how to assist someone who is suicidal
      3. Guidelines on what to do if an employee is experiencing a mental health issue
      4. Guidelines on handling an employee’s return to work following mental-health related absence
      5. Guidelines on managing presentism
      6. Face-to-face workshops with healthcare professionals
      7. Online workshops with healthcare professionals
      8. Links with associations who can provide guidance
      9. Peer-to-peer support
      10. Other. please state.

**D. Anti-stigma Activities**

**Please respond to the following questions in the context of the period before the current pandemic.**

1. **Levels of stigma** (negative attitudes/behaviours around mental health issues)
   1. To what extent have employees the chance to speak openly about their work stress. burnout feelings or mental health problems in the workplace? *(Strongly agree/agree/neither agree nor disagree/disagree/strongly disagree/don’t know)*
   2. To what extent do workplaces?: *(To a large extent/Somewhat/To a small extent/Not at all/Don’t know)*
      1. Have a visible approach to reduce bullying and discrimination related to mental health issues in the workplace.
      2. have policies on sharing information about employees’ mental health problems in order to protect employees’ privacy rights
      3. have policies to protect employees against discrimination and bullying due to their mental health problems
   3. Based on your professional assessment. what is the most common employee attitude regarding openly expressing mental health problems? (*1- Hiding 2 3 4 5 - Full Transparency)*
   4. Based on your professional assessment. what is a manager’s/supervisor’s most common attitude toward employee mental health problems? *(1- rejection 2 3 4 5 – Full acceptance)*
   5. Based on your professional assessment. if someone with a mental health problem openly expresses this in the workplace:
      1. What are the most common risks? *(Open text________________)*
      2. What are the most common benefits?*(Open text________________)*
2. **Activities to reduce stigma** (negative attitudes/behaviours around mental health issues)
   1. To what extent do you think workplaces need further tools to reduce stigma towards mental health problems in the workplace? *(To a large extent/Somewhat/To a small extent/Not at all/Don’t know)*
      1. Printed materials about mental health
      2. Online information materials
      3. Counselling provided or funded by the workplace
      4. Awareness campaigns
      5. Workshops about mental disorders and stigma led by a professional (e.g.. psychologist)
      6. Workshops about mental disorders and stigma led by a person with lived experience
      7. Website about how to reduce stigma in the workplace
      8. E-mail or chat options to discuss stigma
      9. Other: Please state________________
   2. To what degree do you think managers would agree with the following statements about programs to reduce stigma (negative attitudes/behaviours around mental health issues)?

(*To a large extent/Somewhat/To a small extent/Not at all/Don’t know*)

- - 1. Anti-stigma programs can have a positive impact on the wellbeing of employees with mental health problems.
    2. Anti-stigma programs can increase the wellbeing of the entire staff at a workplace
    3. Anti-stigma programs can increase the productivity in the workplace

1. **Acceptability of anti-stigma interventions**
   1. What. if anything. do you consider to be the main barrier when conducting a mental health anti-stigma activity? (open text)

**4. Are you aware of any mental health anti-stigma activities in your country? If yes. please list the name of the program(s):**

**E. GENDER**

1. To what extent do you assess that there are gender differences in terms of help-seeking behaviours related to mental health issues in your area? *(open text)*
2. Do you think there are gender-specific aspects that should be considered when supporting an employee’s mental health? *(open text)*
3. Do you think that specific aspects need to be considered in male dominated workplaces and female dominated workplaces in terms of creating a mentally healthy workplace? *(open text)*

**F. ACCEPTABILITY**

**In this section. we would like you to assess factors which may influence the acceptability of an intervention in the area you are an expert for in terms of:**

1. **Acceptability for managers/supervisors**
   1. To what extent do you think that managers/supervisors might have the following concerns when it comes to implementing mental health interventions within the workplace? (*To a large extent/Somewhat/To a small extent/Not at all/Don’t know)*
      1. Thinking that the workplace is not responsible for employees’ mental health
      2. Thinking that staff will hesitate to participate in interventions in the workplace
      3. Concern about lack of resources for implementation
      4. Concern about employees accessing interventions during work time or using work resources
      5. The workplace is not the appropriate setting for such interventions
      6. Other. please state________________
   2. To what extent do you think the following may influence managers/supervisors when deciding whether or not to implement mental health interventions within the workplace:(*To a large extent/Somewhat/To a small extent/Not at all/Don’t know)*
      1. Information on the economic benefits it could bring to the workplace
      2. Information on the social benefits it could bring to the workplace
      3. Testimonials from managers/supervisors who have implemented mental health interventions and noted positive changes within the business
      4. Scientific research on the benefits of mental health interventions
      5. Simple implementation which requires minimal manager/HR time
      6. Minimal requirement of employee time
      7. Relevance to COVID-19 pandemic
      8. Other. please state
2. **Acceptability for employees**
   1. Based on your experience to what extent do you think the following issues may prevent an employee from participating in mental health interventions within the workplace setting?(*To a large extent/Somewhat/To a small extent/Not at all/Don’t know)*
      1. Concerns about confidentiality
      2. Concerns about discrimination/stigma
      3. Concerns about career progression/job security
      4. Thinking that the workplace should not get involved when employees have mental health problems
      5. Other. please state______________

**3. Acceptability of online tools for interventions aimed at individual employees**

Please rate the following statements about accessing tools online in terms of agreement *(Likert 1-5 strongly agree. agree. neither agree nor disagree. disagree. strongly disagree):*

- - 1. Employees may feel uncomfortable accessing online mental health interventions while being at work
    2. Accessing an online intervention while in the workplace could have negative repercussions for the employee
    3. Employees accessing an online intervention through the workplace could have negative repercussions for the employers/business/SME
    4. Employees in the area have easy access to a computer during working hours
    5. It would be easier for employees to access an intervention through their personal smartphone.

1. Is there anything else you would like to tell us about mental health in the workplace or about implementing activities to support mental health in the workplace? Please add whatever you think is relevant. If you have specific knowledge about one of the three sectors (ICT. health. construction) please provide us with additional information with regard to these sectors and/or if you have specific knowledge about SMEs please provide us with additional knowledge with regard to SMEs (*open text_____________________________________)*
