# Supplementary material for: Supporting employees with mental illness and reducing mental illness-related stigma in the workplace: an expert survey
Source: Eur Arch Psychiatry Clin Neurosci. 2022 Jul 22;273(3):739–53. doi: 10.1007/s00406-022-01443-3 (PMC9305029; doi:10.1007/s00406-022-01443-3)
Supplement: Supplementary file 2 — Supplementary file2 (DOCX 32 KB) [file 406_2022_1443_MOESM2_ESM.docx]

**Online Resource 2. Statistically significant differences in response based on country group.**

**Supporting employees with mental illness and reducing mental illness-related stigma in the workplace: an expert survey**

Bridget Hogg^1,2,3,4^, Ana Moreno-Alcázar^1,2,4^, Mónika Ditta Tóth^5^, Ilinca Serbanescu^6^, Birgit Aust^7^, Caleb Leduc^8,9^, Charlotte Paterson^10^, Fotini Tsantilla^11^, Kahar Abdulla^12^, Arlinda Cerga-Pashoja^13,14^, Johanna Cresswell-Smith^15^, Naim Fanaj^16^, Andia Meksi^17^, Doireann Ni Dhalaigh^9^, Hanna Reich,^18,19^ Victoria Ross^20^, Sarita Sanches^21^, Katherine Thomson^22^, Chantal Van Audenhove^11^, Victor Pérez^,1,2,4,23^, Ella Arensman^8,9,20,22^, Gyorgy Purebl^5^*, Benedikt L. Amann^1,2,4,23,24^ and the MENTUPP consortium

1.Centre Fòrum Research Unit, Institute of Neuropsychiatry and Addiction, Parc de Salut Mar, Barcelona, Spain

2.Mental Health Research Group, Hospital del Mar Medical Research Institute (IMIM), Barcelona, Spain

3.PhD Programme, Dept. of Psychiatry and Forensic Medicine, Universitat Autònoma de Barcelona, Bellaterra, Spain

4.Centro de Investigación Biomédica en Red en Salud Mental (CIBERSAM), Madrid, Spain

5.Institute of Behavioural Sciences, Semmelweis University, Budapest, Hungary

6.Faculty of Psychology and Psychotherapy, University of Heidelberg, Heidelberg, Germany.

7.National Research Centre for the Working Environment, Copenhagen, Denmark

8.School of Public Health, University College Cork, Cork, Ireland

9.National Suicide Research Foundation, Cork, Ireland

10.Nursing, Midwifery and Allied Health Professionals Research Unit, University of Stirling, Stirling, Scotland

11. LUCAS, Center for Care Research and Consultancy, Faculty of Medicine, KU Leuven, Belgium

12.European Alliance Against Depression e.V., Leipzig, Germany

13.Population Health, London School of Hygiene and Tropical Medicine, London, England

14.Global Public Health, Public Health England, United Kingdom

15.Finnish Institute for Health and Welfare (THL)

16.Mental Health Center, Prizren, Kosovo

17.Institute of Public Health, Tirane, Albania.

18.Depression Research Centre of the German Depression Foundation, Department of Psychiatry, Psychosomatic Medicine and Psychotherapy, University Hospital, Goethe University, Frankfurt am Main, Germany

19.German Depression Foundation, Leipzig, Germany

20.Australian Institute for Suicide Research and Prevention, Griffith University, Queensland, Australia

21.Phrenos Center of Expertise for severe mental illness, Utrecht, the Netherlands

22.International Association for Suicide Prevention (IASP), Washington DC, USA

23.Dept. of Psychiatry and Forensic Medicine, Pompeu Fabra University Barcelona, Spain

24.Dept. of Psychiatry and Psychotherapy, Ludwig Maximilian University Hospital Munich, Nussbaumstraße 7, Munich, Germany

*Corresponding author. E-mail: purebl.gyorgy@gmail.com

**Online Resource 2. Statistically significant differences in response based on country group.**

For the item “workplaces have policies on sharing information about employees” the majority of the respondents coming from Western Europe & Australia (19.4%) answered “to a small extent”. whereas the majority of the respondents coming from Central & Eastern Europe (19.4%) answered “not at all”.

|  | | | Policies on sharing information about employees’ MHP in order to protect their privacy rights | | | | Total |
| --- | --- | --- | --- | --- | --- | --- | --- |
|  |  |  | Not at all | To a small extent | Somewhat | To a large extent |  |
|  | Western Europe & Australia | Count | 3 | 12 | 10 | 5 | 30 |
|  |  | % of Total | 4.8% | **19.4%** | 16.1% | 8.1% | 48.4% |
|  | Central & Eastern Europe | Count | 12 | 11 | 4 | 5 | 32 |
|  |  | % of Total | **19.4%** | 17.7% | 6.5% | 8.1% | 51.6% |
| Total | | Count | 15 | 23 | 14 | 10 | 62 |
|  |  | % of Total | 24.2% | 37.1% | 22.6% | 16.1% | 100.0% |

For the item “face-to-face workshops on detecting and managing depression and/or anxiety are a useful material/tool in the workplace” 14% of the respondents coming from Western Europe & Australia disagreed. when only 4% of the respondents coming from Central & Eastern Europe disagreed.

|  | | | Would face-to-face workshops on detecting and managing depression and/or anxiety be a useful material/tool in the workplace? | | Total |
| --- | --- | --- | --- | --- | --- |
|  |  |  | No | Yes |  |
|  | Western Europe & Australia | Count | 7 | 17 | 24 |
|  |  | % of Total | **14.0%** | 34.0% | 48.0% |
|  | Central & Eastern Europe | Count | 2 | 24 | 26 |
|  |  | % of Total | **4.0%** | 48.0% | 52.0% |
| Total | | Count | 9 | 41 | 50 |
|  |  | % of Total | 18.0% | 82.0% | 100.0% |

For the item “interventions based on mindfulness or relaxation therapies are a useful material/tool in the workplace” 10% of the respondents coming from Western Europe & Australia disagreed. when only 2% of the respondents coming from Central & Eastern Europe disagreed.

|  | | | Would interventions based on mindfulness or relaxation therapies be a useful material/tool in the workplace? | | Total |
| --- | --- | --- | --- | --- | --- |
|  |  |  | No | Yes |  |
|  | Western Europe & Australia | Count | 5 | 18 | 23 |
|  |  | % of Total | **10.0%** | 36.0% | 46.0% |
|  | Central & Eastern Europe | Count | 1 | 26 | 27 |
|  |  | % of Total | **2.0%** | 52.0% | 54.0% |
| Total | | Count | 6 | 44 | 50 |
|  |  | % of Total | 12.0% | 88.0% | 100.0% |

The majority (25.9%) of the respondents coming from Western Europe & Australias put item “interventions based on cognitive behavioural therapy. to help change negative thoughts and behaviours” in the third place of the ranking. However. the majority (22.2%) of the respondents coming from Central & Eastern Europe put it in the fourth place.

|  | | | | | | | |
| --- | --- | --- | --- | --- | --- | --- | --- |
|  | | | Interventions based on cognitive behavioural therapy. to help change negative thoughts and behaviours | | | | Total |
|  |  |  | Second place | Third place | Fourth place | Fifth place |  |
|  | Western Europe & Australia | Count | 0 | 7 | 3 | 2 | 12 |
|  |  | % of Total | 0.0% | **25.9%** | 11.1% | 7.4% | 44.4% |
|  | Central & Eastern Europe | Count | 5 | 2 | 6 | 2 | 15 |
|  |  | % of Total | 18.5% | 7.4% | **22.2%** | 7.4% | 55.6% |
| Total | | Count | 5 | 9 | 9 | 4 | 27 |
|  |  | % of Total | 18.5% | 33.3% | 33.3% | 14.8% | 100.0% |

The majority (15.8%) of the respondents coming from Western Europe & Australia put item “interventions based on mindfulness or relaxation techniques” in the first place. when the majority (42.2%) of the respondents coming from Central & Eastern Europe put it in the last places (4^th^ and 5^th^ places).

|  | | | | | | | | |
| --- | --- | --- | --- | --- | --- | --- | --- | --- |
|  | | | Interventions based on mindfulness or relaxation techniques | | | | | Total |
|  |  |  | First place | Second place | Third place | Fourth place | Fifth place |  |
|  | Western Europe & Australia | Count | 3 | 2 | 1 | 2 | 0 | 8 |
|  |  | % of Total | **15.8%** | 10.5% | 5.3% | 10.5% | 0.0% | 42.1% |
|  | Central & Eastern Europe | Count | 0 | 0 | 3 | 4 | 4 | 11 |
|  |  | % of Total | 0.0% | 0.0% | 15.8% | **21.1%** | **21.1%** | 57.9% |
| Total | | Count | 3 | 2 | 4 | 6 | 4 | 19 |
|  |  | % of Total | 15.8% | 10.5% | 21.1% | 31.6% | 21.1% | 100.0% |

For the item “supervisors need face-to-face workshops with healthcare professionals” the majority (23%) of the respondents coming from Western Europe & Australia answered “somewhat”. when the majority (32.8%) of the respondents coming from Central & Eastern Europe answered “to a large extent”.

|  | | | | | | | |
| --- | --- | --- | --- | --- | --- | --- | --- |
|  | | | To what extent do supervisors need face-to-face workshops with healthcare professionals? | | | | Total |
|  |  |  | not at all | to a small extent | somewhat | to a large extent |  |
|  | Western Europe & Australia | Count | 3 | 6 | 14 | 8 | 31 |
|  |  | % of Total | 4.9% | 9.8% | **23.0%** | 13.1% | 50.8% |
|  | Central & Eastern Europe | Count | 3 | 2 | 5 | 20 | 30 |
|  |  | % of Total | 4.9% | 3.3% | 8.2% | **32.8%** | 49.2% |
| Total | | Count | 6 | 8 | 19 | 28 | 61 |
|  |  | % of Total | 9.8% | 13.1% | 31.1% | 45.9% | 100.0% |

For the item “guidelines on managing presenteeism would be useful for supervisors” 9.3% of the respondents coming from Western Europe & Australia disagreed. when none of the respondents (0%) coming from Central & Eastern Europe disagreed.

|  | | | | | |
| --- | --- | --- | --- | --- | --- |
|  | | | Useful for supervisors: guidelines on managing presenteeism | | Total |
|  |  |  | yes | no |  |
|  | Western Europe & Australia | Count | 17 | 4 | 21 |
|  |  | % of Total | 39.5% | **9.3%** | 48.8% |
|  | Central & Eastern Europe | Count | 22 | 0 | 22 |
|  |  | % of Total | 51.2% | **0.0%** | 51.2% |
| Total | | Count | 39 | 4 | 43 |
|  |  | % of Total | 90.7% | 9.3% | 100.0% |
