# Supplementary material for: Supporting employees with mental illness and reducing mental illness-related stigma in the workplace: an expert survey
Source: Eur Arch Psychiatry Clin Neurosci. 2022 Jul 22;273(3):739–53. doi: 10.1007/s00406-022-01443-3 (PMC9305029; doi:10.1007/s00406-022-01443-3)
Supplement: Supplementary file 3 — Supplementary file3 (DOCX 33 KB) [file 406_2022_1443_MOESM3_ESM.docx]

**Online Resource 3. Statistically significant differences based on type of representative.**

**Supporting employees with mental illness and reducing mental illness-related stigma in the workplace: an expert survey**

Bridget Hogg^1,2,3,4^, Ana Moreno-Alcázar^1,2,4^, Mónika Ditta Tóth^5^, Ilinca Serbanescu^6^, Birgit Aust^7^, Caleb Leduc^8,9^, Charlotte Paterson^10^, Fotini Tsantilla^11^, Kahar Abdulla^12^, Arlinda Cerga-Pashoja^13,14^, Johanna Cresswell-Smith^15^, Naim Fanaj^16^, Andia Meksi^17^, Doireann Ni Dhalaigh^9^, Hanna Reich,^18,19^ Victoria Ross^20^, Sarita Sanches^21^, Katherine Thomson^22^, Chantal Van Audenhove^11^, Victor Pérez^,1,2,4,23^, Ella Arensman^8,9,20,22^, Gyorgy Purebl^5^*, Benedikt L. Amann^1,2,4,23,24^ and the MENTUPP consortium

1.Centre Fòrum Research Unit, Institute of Neuropsychiatry and Addiction, Parc de Salut Mar, Barcelona, Spain

2.Mental Health Research Group, Hospital del Mar Medical Research Institute (IMIM), Barcelona, Spain

3.PhD Programme, Dept. of Psychiatry and Forensic Medicine, Universitat Autònoma de Barcelona, Bellaterra, Spain

4.Centro de Investigación Biomédica en Red en Salud Mental (CIBERSAM), Madrid, Spain

5.Institute of Behavioural Sciences, Semmelweis University, Budapest, Hungary

6.Faculty of Psychology and Psychotherapy, University of Heidelberg, Heidelberg, Germany.

7.National Research Centre for the Working Environment, Copenhagen, Denmark

8.School of Public Health, University College Cork, Cork, Ireland

9.National Suicide Research Foundation, Cork, Ireland

10.Nursing, Midwifery and Allied Health Professionals Research Unit, University of Stirling, Stirling, Scotland

11. LUCAS, Center for Care Research and Consultancy, Faculty of Medicine, KU Leuven, Belgium

12.European Alliance Against Depression e.V., Leipzig, Germany

13.Population Health, London School of Hygiene and Tropical Medicine, London, England

14.Global Public Health, Public Health England, United Kingdom

15.Finnish Institute for Health and Welfare (THL)

16.Mental Health Center, Prizren, Kosovo

17.Institute of Public Health, Tirane, Albania.

18.Depression Research Centre of the German Depression Foundation, Department of Psychiatry, Psychosomatic Medicine and Psychotherapy, University Hospital, Goethe University, Frankfurt am Main, Germany

19.German Depression Foundation, Leipzig, Germany

20.Australian Institute for Suicide Research and Prevention, Griffith University, Queensland, Australia

21.Phrenos Center of Expertise for severe mental illness, Utrecht, the Netherlands

22.International Association for Suicide Prevention (IASP), Washington DC, USA

23.Dept. of Psychiatry and Forensic Medicine, Pompeu Fabra University Barcelona, Spain

24.Dept. of Psychiatry and Psychotherapy, Ludwig Maximilian University Hospital Munich, Nussbaumstraße 7, Munich, Germany

*Corresponding author. E-mail: purebl.gyorgy@gmail.com

**Online Resource 3. Statistically significant differences based on type of representative.**

For the item “employees can speak openly about their work stress. burnout feelings or mental health problems in the workplace”. the majority (13.6%) of representatives of construction. health or ICT sector agreed. The majority (8.5%) of the academic experts strongly disagreed and the majority (3.4%) of the representatives of organisations providing services for SMEs or representatives of a group of SMEs disagreed. Finally. the majority (5.1%) of representatives of labour groups and occupational health specialist association groups neither agreed nor disagreed and the majority (8.5%) of other type of representatives disagreed.

|  | | | Extent to which employees can speak openly about their work stress. burnout feelings or mental health problems in the workplace | | | | | Total |
| --- | --- | --- | --- | --- | --- | --- | --- | --- |
|  |  |  | Strongly disagree | Disagree | Neither agree nor disagree | Agree | Strongly agree |  |
| Type of expertise | Represent of construction. health or ICT sector | Count | 2 | 8 | 3 | 8 | 6 | 27 |
|  |  | % of Total | 3.4% | 13.6% | 5.1% | **13.6%** | 10.2% | 45.8% |
|  | Academic expert | Count | 5 | 6 | 3 | 1 | 0 | 15 |
|  |  | % of Total | **8.5%** | 10.2% | 5.1% | 1.7% | 0.0% | 25.4% |
|  | Represent of organisation providing services for SMEs or represent of a group of SMEs | Count | 1 | 2 | 0 | 1 | 0 | 4 |
|  |  | % of Total | 1.7% | **3.4%** | 0.0% | 1.7% | 0.0% | 6.8% |
|  | Labour group. occupational health specialist association group. or advocacy group representative | Count | 1 | 0 | 3 | 0 | 0 | 4 |
|  |  | % of Total | 1.7% | 0.0% | **5.1%** | 0.0% | 0.0% | 6.8% |
|  | Other | Count | 0 | 5 | 3 | 0 | 1 | 9 |
|  |  | % of Total | 0.0% | **8.5%** | 5.1% | 0.0% | 1.7% | 15.3% |
| Total | | Count | 9 | 21 | 12 | 10 | 7 | 59 |
|  |  | % of Total | 15.3% | 35.6% | 20.3% | 16.9% | 11.9% | 100.0% |

For the item “managers have the knowledge and skills to detect a mental health condition in an employee”. the majority (22.2%) of representatives of construction. health or ICT sector answered “to a small extent”. The majority (17.5%) of the academic experts also responded “to a small extent” and the majority (4.8%) of the representatives of organisations providing services for SMEs or representatives of a group of SMEs answered “not at all”. Finally. the majority (4.8%) of representatives of labour groups and occupational health specialist association groups answered “somewhat” and the majority (9.5%) of other type of representatives answered “to a small extent”.

|  | | | | | | | |
| --- | --- | --- | --- | --- | --- | --- | --- |
|  | | | To what extent do supervisors have the knowledge and skills to detect a mental health condition in an employee? | | | | Total |
|  |  |  | not at all | to a small extent | somewhat | to a large extent |  |
| Type of expertise | Represent of construction. health or ICT sector | Count | 6 | 14 | 1 | 10 | 31 |
|  |  | % of Total | 9.5% | **22.2%** | 1.6% | 15.9% | 49.2% |
|  | Academic expert | Count | 2 | 11 | 1 | 1 | 15 |
|  |  | % of Total | 3.2% | **17.5%** | 1.6% | 1.6% | 23.8% |
|  | Represent of organisation providing services for SMEs or represent of a group of SMEs | Count | 3 | 0 | 1 | 0 | 4 |
|  |  | % of Total | **4.8%** | 0.0% | 1.6% | 0.0% | 6.3% |
|  | Labour group. occupational health specialist association group. or advocacy group representative | Count | 2 | 0 | 3 | 0 | 5 |
|  |  | % of Total | 3.2% | 0.0% | **4.8%** | 0.0% | 7.9% |
|  | Other | Count | 1 | 6 | 1 | 0 | 8 |
|  |  | % of Total | 1.6% | **9.5%** | 1.6% | 0.0% | 12.7% |
| Total | | Count | 14 | 31 | 7 | 11 | 63 |
|  |  | % of Total | 22.2% | 49.2% | 11.1% | 17.5% | 100.0% |

For the item “supervisors need guidelines on what to do if an employee is experiencing a mental health issue”. the majority (35%) of representatives of construction. health or ICT sector as well as the majority of academic experts (21.7%) answered “to a large extent”. The majority (3.3%) of the representatives of organisations providing services for SMEs or representatives of a group of SMEs and the majority (5%) of representatives of labour groups and occupational health specialist association groups as well as the majority (1.7%) of other type of representatives answered “somewhat”.

|  | | | | | | | |
| --- | --- | --- | --- | --- | --- | --- | --- |
|  | | | To what extent do supervisors need guidelines on what to do if an employee is experiencing a mental health issue? | | | | Total |
|  |  |  | not at all | to a small extent | somewhat | to a large extent |  |
| Type of expertise | Represent of construction. health or ICT sector | Count | 5 | 1 | 3 | 21 | 30 |
|  |  | % of Total | 8.3% | 1.7% | 5.0% | **35.0%** | 50.0% |
|  | Academic expert | Count | 1 | 0 | 1 | 13 | 15 |
|  |  | % of Total | 1.7% | 0.0% | 1.7% | **21.7%** | 25.0% |
|  | Represent of organisation providing services for SMEs or represent of a group of SMEs | Count | 0 | 1 | 2 | 0 | 3 |
|  |  | % of Total | 0.0% | 1.7% | **3.3%** | 0.0% | 5.0% |
|  | Labour group. occupational health specialist association group. or advocacy group representative | Count | 0 | 0 | 3 | 2 | 5 |
|  |  | % of Total | 0.0% | 0.0% | **5.0%** | 3.3% | 8.3% |
|  | Other | Count | 0 | 0 | 1 | 6 | 7 |
|  |  | % of Total | 0.0% | 0.0% | **1.7%** | 10.0% | 11.7% |
| Total | | Count | 6 | 2 | 10 | 42 | 60 |
|  |  | % of Total | 10.0% | 3.3% | 16.7% | 70.0% | 100.0% |

For the item “supervisors need guidelines on handling an employee’s return following mental-health related absence” the majority (30%) of representatives of construction. health or ICT sector as well as the majority of academic experts (21.7%) answered “to a large extent”. The majority (3.3%) of the representatives of organisations providing services for SMEs or representatives of a group of SMEs and the majority (5%) of representatives of labour groups and occupational health specialist association groups as well as the majority (1.7%) of other type of representatives answered “somewhat”.

|  | | | | | | | |
| --- | --- | --- | --- | --- | --- | --- | --- |
|  | | | To what extent do supervisors need guidelines on handling an employee's return following mental-health related abscence? | | | | Total |
|  |  |  | not at all | to a small extent | somewhat | to a large extent |  |
| Type of expertise | Represent of construction. health or ICT sector | Count | 6 | 1 | 4 | 18 | 29 |
|  |  | % of Total | 10.0% | 1.7% | 6.7% | **30.0%** | 48.3% |
|  | Academic expert | Count | 1 | 0 | 1 | 13 | 15 |
|  |  | % of Total | 1.7% | 0.0% | 1.7% | **21.7%** | 25.0% |
|  | Represent of organisation providing services for SMEs or represent of a group of SMEs | Count | 0 | 1 | 2 | 0 | 3 |
|  |  | % of Total | 0.0% | 1.7% | **3.3%** | 0.0% | 5.0% |
|  | Labour group. occupational health specialist association group. or advocacy group representative | Count | 0 | 0 | 3 | 2 | 5 |
|  |  | % of Total | 0.0% | 0.0% | **5.0%** | 3.3% | 8.3% |
|  | Other | Count | 0 | 0 | 1 | 7 | 8 |
|  |  | % of Total | 0.0% | 0.0% | **1.7%** | 11.7% | 13.3% |
| Total | | Count | 7 | 2 | 11 | 40 | 60 |
|  |  | % of Total | 11.7% | 3.3% | 18.3% | 66.7% | 100.0% |
