# Supplementary material for: Supporting employees with mental illness and reducing mental illness-related stigma in the workplace: an expert survey
Source: Eur Arch Psychiatry Clin Neurosci. 2022 Jul 22;273(3):739–53. doi: 10.1007/s00406-022-01443-3 (PMC9305029; doi:10.1007/s00406-022-01443-3)
Supplement: Supplementary file 4 — Supplementary file4 (DOCX 41 KB) [file 406_2022_1443_MOESM4_ESM.docx]

**Online Resource – Figures**

**Supporting employees with mental illness and reducing mental illness-related stigma in the workplace: an expert survey**

Bridget Hogg^1,2,3,4^, Ana Moreno-Alcázar^1,2,4^, Mónika Ditta Tóth^5^, Ilinca Serbanescu^6^, Birgit Aust^7^, Caleb Leduc^8,9^, Charlotte Paterson^10^, Fotini Tsantilla^11^, Kahar Abdulla^12^, Arlinda Cerga-Pashoja^13,14^, Johanna Cresswell-Smith^15^, Naim Fanaj^16^, Andia Meksi^17^, Doireann Ni Dhalaigh^9^, Hanna Reich,^18,19^ Victoria Ross^20^, Sarita Sanches^21^, Katherine Thomson^22^, Chantal Van Audenhove^11^, Victor Pérez^,1,2,4,23^, Ella Arensman^8,9,20,22^, Gyorgy Purebl^5^*, Benedikt L. Amann^1,2,4,23,24^ and the MENTUPP consortium

1.Centre Fòrum Research Unit, Institute of Neuropsychiatry and Addiction, Parc de Salut Mar, Barcelona, Spain

2.Mental Health Research Group, Hospital del Mar Medical Research Institute (IMIM), Barcelona, Spain

3.PhD Programme, Dept. of Psychiatry and Forensic Medicine, Universitat Autònoma de Barcelona, Bellaterra, Spain

4.Centro de Investigación Biomédica en Red en Salud Mental (CIBERSAM), Madrid, Spain

5.Institute of Behavioural Sciences, Semmelweis University, Budapest, Hungary

6.Faculty of Psychology and Psychotherapy, University of Heidelberg, Heidelberg, Germany.

7.National Research Centre for the Working Environment, Copenhagen, Denmark

8.School of Public Health, University College Cork, Cork, Ireland

9.National Suicide Research Foundation, Cork, Ireland

10.Nursing, Midwifery and Allied Health Professionals Research Unit, University of Stirling, Stirling, Scotland

11. LUCAS, Center for Care Research and Consultancy, Faculty of Medicine, KU Leuven, Belgium

12.European Alliance Against Depression e.V., Leipzig, Germany

13.Population Health, London School of Hygiene and Tropical Medicine, London, England

14.Global Public Health, Public Health England, United Kingdom

15.Finnish Institute for Health and Welfare (THL)

16.Mental Health Center, Prizren, Kosovo

17.Institute of Public Health, Tirane, Albania.

18.Depression Research Centre of the German Depression Foundation, Department of Psychiatry, Psychosomatic Medicine and Psychotherapy, University Hospital, Goethe University, Frankfurt am Main, Germany

19.German Depression Foundation, Leipzig, Germany

20.Australian Institute for Suicide Research and Prevention, Griffith University, Queensland, Australia

21.Phrenos Center of Expertise for severe mental illness, Utrecht, the Netherlands

22.International Association for Suicide Prevention (IASP), Washington DC, USA

23.Dept. of Psychiatry and Forensic Medicine, Pompeu Fabra University Barcelona, Spain

24.Dept. of Psychiatry and Psychotherapy, Ludwig Maximilian University Hospital Munich, Nussbaumstraße 7, Munich, Germany

*Corresponding author. E-mail: purebl.gyorgy@gmail.com

**Online Resource Fig. 1 Breakdown of specific expertise by country type**

**Online Resource Fig. 2 Current level of unmet need for programmes to prevent and treat mental health difficulties in employees by % of experts**

**Online Resource Fig. 3 Open text responses regarding risks of employees openly expressing mental health problems, by % of experts who provided each response.**

**Online Resource Fig. 4 Open text responses regarding benefits of employees openly expressing mental health problems. by % of experts who provided each response.**

**Online Resource Fig. 5 Open text responses regarding barriers to implementing anti-stigma programmes. by % of experts who provided each response.**
