# Supplementary material for: Supporting employees with mental illness and reducing mental illness-related stigma in the workplace: an expert survey
Source: Eur Arch Psychiatry Clin Neurosci. 2022 Jul 22;273(3):739–53. doi: 10.1007/s00406-022-01443-3 (PMC9305029; doi:10.1007/s00406-022-01443-3)
Supplement: Supplementary file 5 — Supplementary file5 (DOCX 46 KB) [file 406_2022_1443_MOESM5_ESM.docx]

**Supporting employees with mental illness and reducing mental illness-related stigma in the workplace: an expert survey**

Bridget Hogg^1,2,3,4^, Ana Moreno-Alcázar^1,2,4^, Mónika Ditta Tóth^5^, Ilinca Serbanescu^6^, Birgit Aust^7^, Caleb Leduc^8,9^, Charlotte Paterson^10^, Fotini Tsantilla^11^, Kahar Abdulla^12^, Arlinda Cerga-Pashoja^13,14^, Johanna Cresswell-Smith^15^, Naim Fanaj^16^, Andia Meksi^17^, Doireann Ni Dhalaigh^9^, Hanna Reich,^18,19^ Victoria Ross^20^, Sarita Sanches^21^, Katherine Thomson^22^, Chantal Van Audenhove^11^, Victor Pérez^,1,2,4,23^, Ella Arensman^8,9,20,22^, Gyorgy Purebl^5^*, Benedikt L. Amann^1,2,4,23,24^ and the MENTUPP consortium

1.Centre Fòrum Research Unit, Institute of Neuropsychiatry and Addiction, Parc de Salut Mar, Barcelona, Spain

2.Mental Health Research Group, Hospital del Mar Medical Research Institute (IMIM), Barcelona, Spain

3.PhD Programme, Dept. of Psychiatry and Forensic Medicine, Universitat Autònoma de Barcelona, Bellaterra, Spain

4.Centro de Investigación Biomédica en Red en Salud Mental (CIBERSAM), Madrid, Spain

5.Institute of Behavioural Sciences, Semmelweis University, Budapest, Hungary

6.Faculty of Psychology and Psychotherapy, University of Heidelberg, Heidelberg, Germany.

7.National Research Centre for the Working Environment, Copenhagen, Denmark

8.School of Public Health, University College Cork, Cork, Ireland

9.National Suicide Research Foundation, Cork, Ireland

10.Nursing, Midwifery and Allied Health Professionals Research Unit, University of Stirling, Stirling, Scotland

11. LUCAS, Center for Care Research and Consultancy, Faculty of Medicine, KU Leuven, Belgium

12.European Alliance Against Depression e.V., Leipzig, Germany

13.Population Health, London School of Hygiene and Tropical Medicine, London, England

14.Global Public Health, Public Health England, United Kingdom

15.Finnish Institute for Health and Welfare (THL)

16.Mental Health Center, Prizren, Kosovo

17.Institute of Public Health, Tirane, Albania.

18.Depression Research Centre of the German Depression Foundation, Department of Psychiatry, Psychosomatic Medicine and Psychotherapy, University Hospital, Goethe University, Frankfurt am Main, Germany

19.German Depression Foundation, Leipzig, Germany

20.Australian Institute for Suicide Research and Prevention, Griffith University, Queensland, Australia

21.Phrenos Center of Expertise for severe mental illness, Utrecht, the Netherlands

22.International Association for Suicide Prevention (IASP), Washington DC, USA

23.Dept. of Psychiatry and Forensic Medicine, Pompeu Fabra University Barcelona, Spain

24.Dept. of Psychiatry and Psychotherapy, Ludwig Maximilian University Hospital Munich, Nussbaumstraße 7, Munich, Germany

*Corresponding author. E-mail: purebl.gyorgy@gmail.com

### Online Resource Table 1. Breakdown of expert category and specific expert skills by country.

| Expert category (one response per expert) | Total | Albania | Australia | Finland | Germany | Hungary | Ireland | Kosovo | Spain | Netherlands |
| --- | --- | --- | --- | --- | --- | --- | --- | --- | --- | --- |
| Labour group, occupational health specialist association group, or advocacy group representative | 5 | 1 | 0 | 1 | 0 | 0 | 1 | 0 | 1 | 1 |
| Academic expert | 15 | 4 | 1 | 3 | 0 | 0 | 2 | 0 | 3 | 2 |
| Representative of an organisation providing services for SMEs or representing a group of SMEs. | 4 | 0 | 0 | 0 | 0 | 3 | 0 | 1 | 0 | 0 |
| Representative of an organisation representing the construction, health or information and communication technologies (ICT) sector. | 32 | 8 | 1 | 0 | 4 | 5 | 0 | 6 | 5 | 3 |
| Other | 9 | 3 | 0 | 2 | 0 | 2 | 0 | 1 | 0 | 1 |
| Specific expert experience (experts may select more than one response) | Total | Albania | Australia | Finland | Germany | Hungary | Ireland | Kosovo | Spain | Netherlands |
| Construction industry | 10 | 1 | 2 | 0 | 0 | 1 | 3 | 1 | 1 | 1 |
| Health care sector | 30 | 6 | 0 | 3 | 3 | 3 | 3 | 4 | 4 | 4 |
| IT and communication | 16 | 5 | 0 | 0 | 2 | 6 | 1 | 1 | 1 | 0 |
| SME | 11 | 2 | 0 | 0 | 1 | 3 | 1 | 1 | 2 | 1 |
| Mental health in SMEs | 12 | 3 | 1 | 1 | 0 | 1 | 1 | 1 | 2 | 2 |
| My experience is general and not related to any of these sectors | 10 | 2 | 0 | 3 | 0 | 2 | 0 | 0 | 2 | 1 |

### Online Resource Table 2. Knowledge and skills of managers by *n* and % of experts.

| Knowledge and skills of managers to | To a large extent  (4) | Some-what  (3) | To a small extent  (2) | Not at all  (1) | Don’t know | M  (IQR) |
| --- | --- | --- | --- | --- | --- | --- |
| Detect a mental health condition in an employee | 11  16.9% | 7  10.8% | 31  47.7% | 14  21.5% | 2  3.1% | 2  (1) |
| Have a conversation about employee’s mental health condition | 9  13.8% | 11  16.9% | 29  44.6% | 16  24.6% | 0  0% | 2  (2) |
| Make adjustments to facilitate job retention or return to work | 9  13.8% | 11  16.9% | 27  41.5% | 14  21.5% | 4  6.2% | 2  (1) |

Key. M: Median; IQR: Interquartile Range.

### Online Resource Table 3. Needs of managers by *n* and % of experts.

| To what extent need managers the following tools or materials | To a large extent  (4) | Some-what  (3) | To a small extent  (2) | Not at all  (1) | Don’t know | M  (IQR) | Would this be useful?  Yes | Would this be useful?  No |
| --- | --- | --- | --- | --- | --- | --- | --- | --- |
| Materials providing information about depression or anxiety and how to cope | 30  46.2% | 18  27.7% | 6  9.2% | 5  7.7% | 2  3.1% | 4  (1) | 43  66.2% | 5  7.7% |
| Materials providing information about suicide and how to access help | 24  36.9% | 17  26.2% | 9  13.8% | 7  10.8% | 3  4.6% | 3  (2) | 42  64.6% | 6  9.2% |
| Guidelines on what to do if an employee is experiencing mental health issues | 42  64.6% | 10  15.4% | 2  3.1% | 6  9.2% | 2  3.1% | 4  (1) | 44  67.7% | 5  7.7% |
| Guidelines on handling an employee’s return following mental health related absence | 40  61.5% | 11  16.9% | 2  3.1% | 7  10.8% | 2  3.1% | 4  (1) | 42  64.6% | 5  7.7% |
| Guidelines on managing presenteeism | 28  43.1% | 16  24.6% | 4  6.2% | 7  10.8% | 7  10.8% | 4  (1) | 39  60% | 4  6.2% |
| Face-to-face workshops with healthcare professionals | 28  43.1% | 19  29.2% | 8  12.3% | 6  9.2% | 1  1.5% | 3  (1) | 42  64.6% | 5  7.7% |
| Online workshops with healthcare professionals | 23  35.4% | 19  29.2% | 8  12.3% | 9  13.8% | 2  3.1% | 3  (2) | 36  55.4% | 9  13.8% |
| Guidance from linked associations | 23  35.4% | 21  32.3% | 10  15.4% | 7  10.8% | 0  0% | 3  (2) | 37  56.9% | 8  12.3% |
| Peer-to-peer support | 27  41.5% | 18  27.7% | 9  13.8% | 6  9.2% | 1  1.5% | 3  (2) | 41  63.1% | 4  6.2% |

Key. M: Median; IQR: Interquartile Range.

### Online Resource Table 4 Assessment of levels of stigma by *n* and % of experts.

| Level of stigma | Strongly agree  (5) | Agree  (4) | Neutral  (3) | Disagree  (2) | Strongly disagree (1) | Don’t know | M  (IQR) |
| --- | --- | --- | --- | --- | --- | --- | --- |
| Employees can speak openly about their work stress. burnout feelings or mental health problems | 7  10.8% | 10  15.4% | 12  18.5% | 21  32.3% | 9  13.8% | 6  9.2% | 2  (2) |

Key. M: Median; IQR: Interquartile Range.

### Online Resource Table 5. Assessment of the extent of current measures to reduce mental health-related discrimination in the workplace by *n* and % of experts.

| To what extent do workplaces | To a large extent  (4) | Some-what  (3) | To a small extent  (2) | Not at all  (1) | Don’t know | M  (IQR) |
| --- | --- | --- | --- | --- | --- | --- |
| Have a visible approach to reduce bullying and discrimination related to mental health issues in the workplace | 7  10.8% | 14  21.5% | 29  44.6% | 10  15.4% | 5  7.7% | 2  (1) |
| Have policies on sharing information about employees’ mental health problems in order to protect employees’ privacy rights | 10  15.4% | 14  21.5% | 23  35.4% | 15  23.1% | 0  0% | 2  (1) |
| Have policies to protect employees against discrimination and bullying due to their mental health problems | 6  9.2% | 15  23.1% | 25  38.5% | 16  24.6% | 0  0% | 2  (2) |

 Key. M: Median; IQR: Interquartile Range.

### Online Resource Table 6. Perception of managers’ views regarding anti-stigma programmes by *n* and % of experts.

| Degree to which managers would agree with following statements about anti-stigma programmes | To a large extent (4) | Some-what  (3) | To a small extent (2) | Not at all  (1) | Don’t know | M  (IQR) |
| --- | --- | --- | --- | --- | --- | --- |
| Anti-stigma programmes have a positive impact | 22  33.8% | 27  41.5% | 14  21.5% | 0  0% | 2  3.1% | 3  (1) |
| Anti-stigma programmes can increase wellbeing | 24  36.9% | 24  36.9% | 13  20% | 1  1.5% | 3  4.6% | 3  (1) |
| Anti-stigma programmes can increase productivity | 23  35.4% | 23  35.4% | 13  20% | 3  4.6% | 3  4.6% | 3  (2) |

Key. M: Median; IQR: Interquartile Range.

**Online Resource Table 7. List of Anti-Stigma activities mentioned**

| Name of anti-stigma programme | Country where expert who mentioned it is based |
| --- | --- |
| ‘I AM WHOLE’-campaign <https://www.whole.org.uk/> | Albania |
| <https://www.who.int/westernpacific/news/events/world-health-day> | Albania, Finland, Germany |
| MATES in Construction https://mates.org.au/ | Australia |
| Accenture Allies Programme <https://www.accenture.com/gb-en/company-accenture-allies-programmes> | Finland |
| Mielekäs työ by Mielenterveyspooli (translation: Mental Health Pool): <https://mielenterveyspooli.fi/> | Finland |
| Hyvän mielen työpaikka (translation: Brain Work) <https://www.ttl.fi/oppimateriaalit/en/> | Finland |
| Työkyvyn tuki edistää mielenterveyttä ja työhön osallistumista (translation: Support for work ability promotes mental health and participation in work) <https://tietokayttoon.fi/julkaisu?pubid=36401> | Finland |
| World Suicide Prevention Day | Finland |
| Programmes run by Finnish Institute for Occupational Health | Finland |
| Programmes run by German Depression Foundation | Germany |
| See change by Green Ribbon <https://seechange.ie/green-ribbon/> | Ireland |
| Anti-stigma programmes for people with HIV and drug abusers | Kosovo |
| Samen Sterk zonder Stigma (translation: Strong Together without Stigma): <https://www.samensterkzonderstigma.nl/> | The Netherlands |
| Per la Salut Mental, dóna la cara by Obertament (translation: for mental health, show your face): <https://obertament.org/ca> | Spain |
| DIXIT TV: (Social Services Documentation Centre) <https://dixit.gencat.cat/en/01dixit/01que_es/> | Spain |
| Confederación Salud Mental España (translation: Spain mental health confederation: consaludmental.org | Spain |
